# Epidemiology and Antimicrobial-Resistant Genes of Family Staphylococcaceae in *Musca domestica*: Case Studies from Chicken Farm, Pig Farms, and Residential Areas in Southern Thailand

**DOI:** 10.3390/insects17050461

**Published:** 2026-04-28

**Authors:** Narin Sontigun, Nattharee Thanawan, Punpichaya Fungwithaya

**Affiliations:** 1 Office of Administrative Interdisciplinary Program on Agricultural Technology (OAIPAT), School of Agricultural Technology, King Mongkut’s Institute of Technology Ladkrabang, Bangkok 10520, Thailand; narin.so@kmitl.ac.th; 2 Microbiology Laboratory, Division of Clinical Pathology, Army Institute of Pathology, Phramongkutklao Medical Center, Royal Thai Army Medical Department, Bangkok 10400, Thailand; nattharee.th@gmail.com; 3 Department of Microbiology, Faculty of Medicine Siriraj Hospital, Mahidol University, Bangkok 10700, Thailand

**Keywords:** epidemiology, *Musca domestica*, resistant genes, Staphylococcaceae, Thailand

## Abstract

The major Staphylococcaceae family is recognized as opportunistic pathogens colonizing human and animal skin, mucous membranes, and environments. *Musca domestica,* the house fly, plays a role in the transmission of AMR bacteria. This study focused on examining the epidemiology and antimicrobial-resistant genes of the family Staphylococcaceae in *M. domestica* through metagenomic analysis, using samples collected from three animal farms and two residential areas in southern Thailand. Fifty *M. domestica* were collected from five places surrounding Walailak University, including one chicken farm (CF1), two pig farms (PF2 and PF3), and two residential areas (H1 and H2). All samples were dispatched for analysis using shotgun metagenomic sequencing and analyzed using FastQC, MultiQC, FASTQ, MEGAHIT, QUAST, ABRicate, AMRFinderPlus, ResFinder, ARG-ANNOT, MEGARES, PlasmidFinder, VFDB, Kraken2, Krona and Python. Our findings describe the taxonomic composition of Staphylococcaceae taxa in *M. domestica* from different environments; the representation of the family Staphylococcaceae in CF1, PF2, PF3, H1, and H2 was recorded at 2%, 0.7%, 0.2%, 0.2%, and 2% of this phylum, respectively. The average populations discovered were *Staphylococcus* (37.4%), *Mammaliicoccus* (17.4%), and *Macrococcus* (10.3%), respectively. Trimethoprim-resistant genes (*dfrG* and *dfrE*) were found only in CF1, PF2, and H1. Interestingly, fosfomycin-resistant genes were found only in *M. domestica* within residential areas. Our findings pertain to the Staphylococcaceae population in *M. domestica* within residential areas, which exhibited varying multidrug-resistance genes, particularly those resistant to fosfomycin.

## Simple Summary

*Musca domestica* was recognized as a carrier and distributor of the pathogen, while most members of the family Staphylococcaceae are involved in the transmission of significant zoonotic pathogens. Therefore, this investigation concentrated on the metagenomic analysis of data obtained from three animal farms and two residential areas in Southern Thailand to investigate the epidemiology and antimicrobial-resistant genes of the family Staphylococcaceae in *M. domestica*. The variable of species was shared between CF1 and H1 more than the other areas. *Staphylococcus* was the most prevalent species (37.4%), with approximately 1% of these bacteria being detected in all locations. Residential areas were identified as the most significant source of drug-resistant genes, particularly fosfomycin, which poses a serious public health risk due to the potential for these genes to spread to both humans and animals.

## 1. Introduction

The family Staphylococcaceae comprises three primary genera, including *Macrococcus*, *Mammaliicoccus,* and *Staphylococcus* [1,2,3]. The typical structure of this family is characterized by Gram-positive, non-spore-forming, spherical or coccoid cells that exhibit non-motility. These microorganisms are commonly identified as grape-like clusters and can thrive in both aerobic and facultatively anaerobic environments. The main biochemical test shows catalase positivity and displays variable oxidase activity [1,4]. The genera *Macrococcus* and *Staphylococcus* are divided by their DNA G+C content, which is 70 mol% and 33–40 mol%, respectively [5]. *Mammaliicoccus*, the newest genus in this family, was reclassified from *Staphylococcus* [1]. Most of this family, recognized as opportunistic pathogens, is frequently located in diverse habitats such as human and animal skin, mucous membranes, and environments [4,6,7]. These pathogens are responsible for skin infections, pyoderma, and nosocomial infections in both veterinary and human healthcare settings [8,9,10]. Horizontal gene transfer (HGT) is a famous mechanism of antimicrobial resistance (AMR) to interchange antimicrobial resistance genes (ARGs) within this family. Staphylococcal cassette chromosome *mec* (SCC*mec*) is the mobile genetic element that can exchange resistant genes among the genus *Staphylococcus* [11,12]. Multidrug-resistant pathogens contribute to nosocomial infections in both humans and animals in hospitals [13]. Additionally, veterinary hospitals experienced recurrent infections due to environmental contamination involving this pathogen [14,15,16].

*Musca domestica*, commonly known as the house fly, is a prevalent insect found in various environments. These flies not only inhabit the human environment but are also remarkably adaptable to various ecological niches, including urban, agricultural, and waste-heavy settings. Given their size and behavior, these organisms serve as carriers for a range of pathogens, such as bacteria, viruses, and fungi, which further heighten health risks within communities [17,18]. House flies act as mechanical transmitters of microbes, especially multi-drug-resistant pathogens, within thirteen miles through their external body surfaces (legs, wings, thorax) and mouthparts, as well as defecation and regurgitation during feeding [17,19]. The feeding behavior of *M. domestica* plays a crucial role in the transmission of various microbial diversities, including antibiotic-resistant bacteria, to environments such as hospitals, farms, and waste disposal sites [20,21]. As the issue of AMR gains more attention, these flies are being acknowledged more frequently as environmental reservoirs and vectors for the spread of ARGs [22]. Examining their role in the proliferation of AMR is critical to understanding transmission dynamics and formulating targeted mitigation strategies to tackle this global challenge.

It is essential to investigate the family Staphylococcaceae in *M. domestica* around animal farms and residential areas to monitor and control the spread of zoonotic infections [23]. In Thailand, reports indicate that *M. domestica* carries bacteria in the northern and northeastern regions [24,25]. The family Staphylococcaceae exhibited the highest population density in the northern region. Currently, there are no documented findings concerning the population of the family Staphylococcaceae in *M. domestica* from the southern region through metagenomic analysis. This study focused on examining the epidemiology and antimicrobial-resistant genes of the family Staphylococcaceae on the external body surfaces of *M. domestica* through metagenomic analysis, using data collected from three animal farms and two residential areas in Southern Thailand.

## 2. Materials and Methods

### 2.1. Ethical Statements

The current study protocol was approved by the Institutional Animal Care and Use Committee of Walailak University (approved number: WU-ACUC-65017).

### 2.2. Sample Collection and DNA Extraction

**Figure 1:**
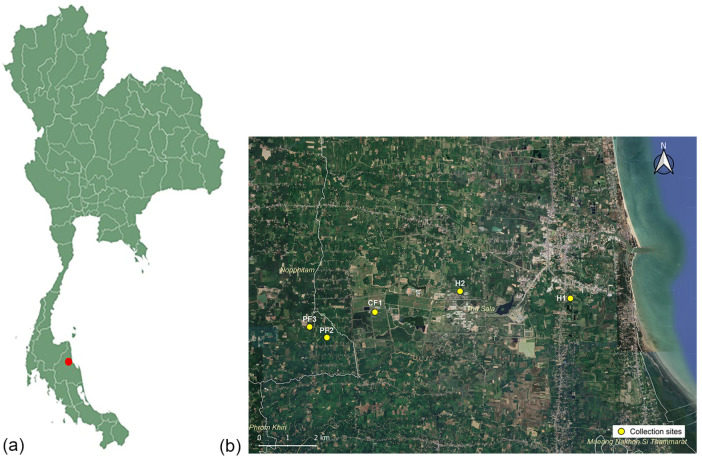
A map showing the locations for sample collection in Nakhon Si Thammarat, southern Thailand. (**a**) A red circle indicates the location of Walailak University based on the Thailand map imagery from Simplemaps. Available online: https://simplemaps.com/svg/country/th (accessed on 22 April 2026). (**b**) Yellow circles indicate the locations of five collection sites surrounding Walailak University, including one chicken farm (CF1), two pig farms (PF2 and PF3), and two residential areas (H1 and H2), based on the satellite imagery from Google Earth. Available online: https://earth.google.com (accessed on 23 April 2025).

Five places surrounding Walailak University were selected as collection sites from August to September 2022, including one chicken farm (CF1; N 8°38′34.1052″, E 99°52′4.4868″), two pig farms (PF2; N 8°38′4.8696″, E 99°51′9.0756″ and PF3; N 8°38′17.1312″, E 99°50′49.1064″), and two residential areas (H1; N 8°38′49.9632″, E 99°55′49.98″ and H2; N 8°38′58.1712″, E 99°53′42.7236″) (Figure 1). All locations are within inter-site distances of less than 10 km, according to several studies [26,27,28], indicating that house flies can travel varying maximum flight distances ranging from 7 to 32 km. The first residential area (H1) is positioned 30 m from the chicken slaughterhouse, while the second one (H2) is located 5 m from the university cafeteria. Ten *M. domestica* were collected from each site using sweep nets. They were placed in individual sterile 15 mL vials, sent to the laboratory within one hour, and then frozen for 15 min at −20 °C for euthanasia. All flies were confirmed as *M. domestica* using a taxonomic key of Tumrasvin and Shinonaga [29] under a stereomicroscope (Olympus, Tokyo, Japan) before placing them in 1 mL tryptic soy broth (TSB) and subjecting them to vigorous vortexing for 1 min. After each fly was removed, the remaining suspension was centrifuged at 10,000× *g* for 10 min at 4 °C. Subsequently, the supernatant was discarded, and the DNA extraction procedure was conducted using the Presto™ DNA/RNA Extraction Kit (Geneaid, New Taipei City, Taiwan), in accordance with the manufacturer’s guidelines. Five pooled DNA samples were dispatched to Novogene for analysis using shotgun metagenomic sequencing (Novogene, Helios, Singapore). The genomic DNA libraries were generated using the VAHTS Universal DNA Library Prep Kit for Illumina V3 (Vazyme, Nanjing, China) in a non-PCR-free manner, with a target insert size of 350 bp. The sequencing was carried out on Illumina NovaSeq 6000 equipment (Illumina, San Diego, CA, USA) using paired-end 150 bp (PE150) mode. To ensure the integrity of our downstream analysis, the resulting raw reads with fastp provide high-quality clean data. This filtering phase comprised cutting adaptor sequences and removing reads with more than 10% unclear bases (N). Low-quality reads with more than half of the bases were deleted at a Q-score of 5 or lower, ensuring that only credible sequences were used for future bioinformatic study.

### 2.3. Bioinformatics Analysis Pipeline

Shotgun metagenomic sequencing was performed on five samples, generating an average of ~20 million reads per sample. Bioinformatics analysis was performed on the Galaxy platform using a standardized and reproducible pipeline. Raw sequencing data were assessed for quality using FastQC (v0.12.1) [30], and results were aggregated with MultiQC (v1.9) [31]. Read preprocessing, including quality filtering and adapter trimming, was conducted using fastp (v0.20.1) with default parameters; no additional trimming was required as adapter sequences had been removed prior to analysis. High-quality reads were assembled de novo into contigs using MEGAHIT (v0.4.8.0), and assembly quality was evaluated using QUAST (v5.0.2) [32,33]. Functional annotation of contigs was performed using ABRicate (v1.0.1) [34] to identify antimicrobial resistance and virulence-associated genes based on multiple reference databases, including NCBI AMRFinderPlus, CARD, ResFinder, ARG-ANNOT, MEGARES, PlasmidFinder, and VFDB. Taxonomic classification was conducted using Kraken2 (v2.1.1) with the standard database (released 7 June 2022), employing an exact k-mer matching approach with a lowest common ancestor (LCA) algorithm under default parameters. Taxonomic profiles were visualized using Krona (v2.7.1) to enable hierarchical exploration of microbial community composition [35,36].

### 2.4. Data Processing

All sequencing reads from the five sample groups (CF1, H1, H2, PF2, and PF3) were used for taxonomic classification using Kraken2 (v2.1.2) with the standard database. The classification tables were created using Python (v3.10) [37] with the pandas library to generate taxonomic profiles for each group, including only taxa with non-zero counts. Taxa sets were constructed for each group, and the number of taxa in all possible intersections was calculated using set operations [38]. The distribution of shared and unique taxa across the groups was visualized using an UpSet plot, which provides an efficient representation of multiple set intersections.

### 2.5. Statistical Analysis

The metagenomic reads were taxonomically categorized using Kraken2 software (version 2.1.1; Johns Hopkins University, Baltimore, USA) with the standard database (released 7 June 2022) to determine and quantify the microbial composition of each sample. The relative abundance of viral taxa was visualized using Krona tools, which automatically calculate the percentage of each taxon based on the outcomes of the hierarchical classification [35,36]. Descriptive analysis was used for the description of the population of members in the family Staphylococcaceae via Jamovi (version 2.3; jamovi project, Sydney, Australia) [38].

## 3. Results

### 3.1. Diversity of Species in M. domestica in Five Areas

**Figure 2:**
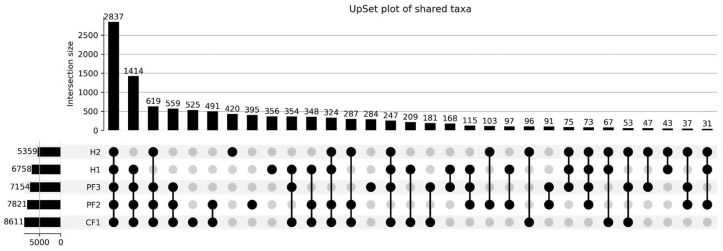
The Venn diagram shows shared and unique taxa among five samples. Each circle represents a different group: CF1, H1, H2, PF2, and PF3. Taxa were filtered to include only those with non-zero counts in the respective samples. This figure visually summarizes the distribution and overlaps of taxa across all five groups.

The diversity and distribution of bacterial taxa associated with *M. domestica* collected from five sampling locations (CF1, H1, H2, PF2, and PF3) were analyzed using an UpSet plot and shown in Figure 2 and Table S1 (Supplementary File). This approach enabled the visualization of shared and unique taxa across multiple groups. A total of 2837 taxa were identified as core microbiota shared among all five groups, indicating the presence of a substantial common bacterial community across different sampling locations. In addition, 1414 taxa were shared among four groups (CF1, H1, PF2, and PF3), further supporting the similarity in microbial composition among these environments.

Unique taxa were also observed in each group, with CF1 exhibiting the highest number (525 taxa), followed by H2 (420 taxa), PF2 (395 taxa), H1 (356 taxa), and PF3 (284 taxa). These findings suggest the presence of location-specific microbial signatures. When considering lower-order intersections, the largest pairwise overlap was observed between CF1 and PF2 (491 taxa), indicating a relatively closer similarity in bacterial composition between these two groups compared to other combinations.

### 3.2. Population of Family Staphylococcaceae

**Figure 3:**
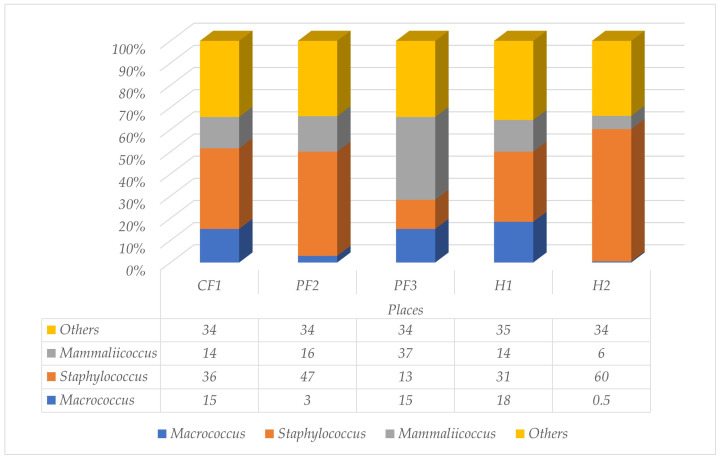
Percentages of different genera within the family Staphylococcaceae isolated from *M. domestica* collected across one chicken farm (CF1), two pig farms (PF2 and PF3), and two residential areas (H1 and H2) around Walailak University.

In Southern Thailand, a total of 50 houseflies were collected from three animal farms and two residential areas. Approximately 93.8% of the five different locations exhibited the presence of bacteria. Firmicutes constituted 14–54% of the bacterial population identified in this study. The representation of the family Staphylococcaceae in CF1, PF2, PF3, H1, and H2 was recorded at 2%, 0.7%, 0.2%, 0.2%, and 2% of this phylum, respectively. The four principal genera of this family, including *Macrococcus*, *Staphylococcus*, *Mammaliicoccus*, and others, were shown in this study. The average populations discovered were *Staphylococcus* (37.4%), *Mammaliicoccus* (17.4%), and *Macrococcus* (10.3%), respectively. The flies that lived near the university cafeteria (H2) showed the highest population of *Staphylococcus* spp. (60%), while the percentage of *Macrococcus* spp. was lower than in the other places (0.5%). Figure 3 and Figures S1–S5 (Supplementary File) illustrated the proportion of each species within the family Staphylococcaceae identified in *M. domestica*.

### 3.3. Population of Staphylococcus spp.

**Figure 4:**
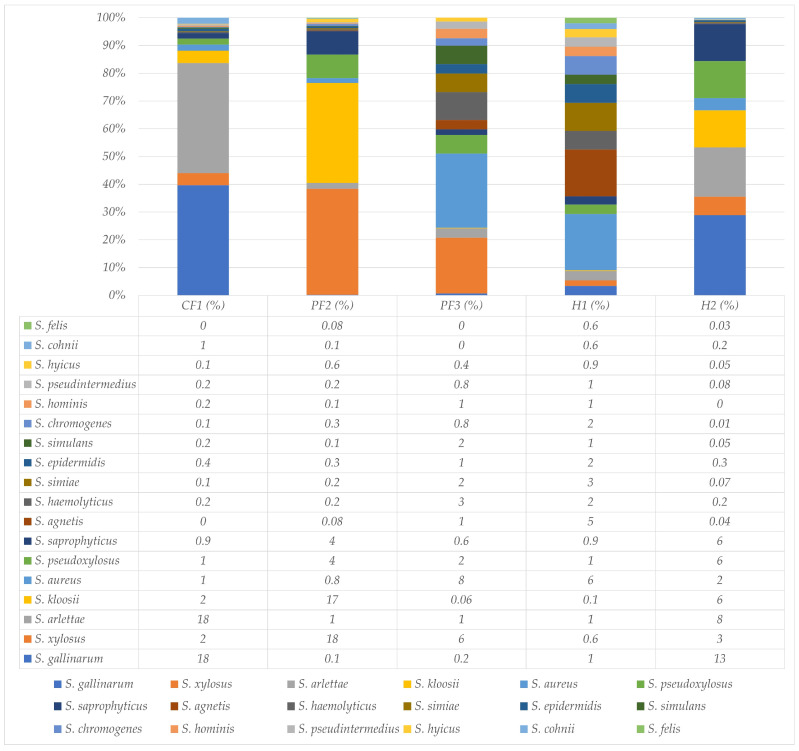
Percentages of species in the genus *Staphylococcus* isolated from *M. domestica* collected across one chicken farm (CF1), two pig farms (PF2 and PF3), and two residential areas (H1 and H2) around Walailak University.

The proportion of significant *Staphylococcus* spp. within the genus *Staphylococcus* was illustrated in Figure 4. The percentage of *Staphylococcus* spp. in CF1, PF2, PF3, H1, and H2 was 36, 47, 13, 31, and 60, respectively. The average of coagulase-negative staphylococci (CoNS), coagulase-positive staphylococci (CoPS), and other staphylococci was 35.1%, 4.02%, and 60.88%, respectively. A total of 18 significant populations of *Staphylococcus* spp. were employed for analysis. Overall, *Staphylococcus (S.) gallinarum*, which was CoNS, showed the highest identification rate among *Staphylococcus* spp. at 6.49% of all places, especially CF1 and H2. The highest population of *Staphylococcus* spp. was discovered on the pig farm (PF3; 8% of *Staphylococcus* spp.); on the other hand, the lowest population of this pathogen was found on the other pig farm (PF2; 0.8% *Staphylococcus* spp.); additionally, *S. pseudintermedius* was discovered in all places (approximately 0.46%).

### 3.4. Population of Mammaliicoccus spp.

**Figure 5:**
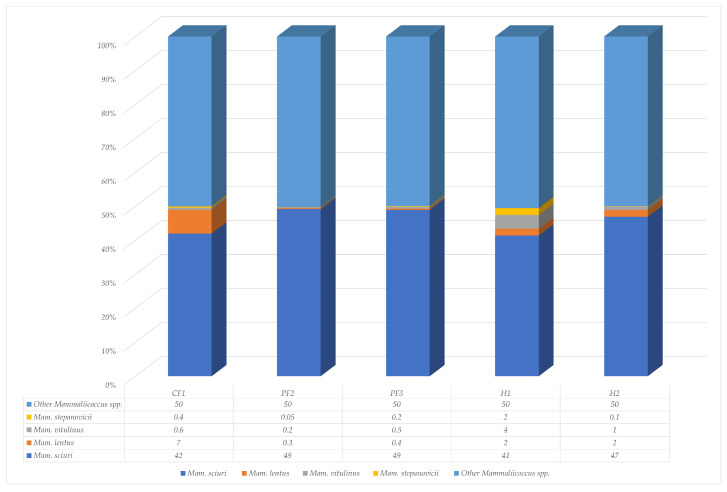
Percentages of species in the genus *Mammaliicoccus* isolated from *M. domestica* collected across one chicken farm (CF1), two pig farms (PF2 and PF3), and two residential areas (H1 and H2) around Walailak University.

According to Figure 5, the four primary *Mammaliicoccus* spp. were *Mammaliicoccus* (*Mam.*) *sciuri*, *Mam. lentus*, *Mam. vitulinus*, and *Mam. stepanovicii*. The percentage of *Mammaliicoccus* spp. in CF1, PF2, PF3, H1, and H2 was 14, 16, 37, 14, and 16, respectively. The most frequently recognized *Mammaliicoccus* species were *Mam. sciuri* (45.6%), *Mam. lentus* (2.34%), *Mam. vitulinus* (1.26%), and *Mam. stepanovicii* (0.55%), respectively. The highest percentage of *Mam. sciuri* was found on two pig farms at 49%. The largest population of *Mam. lentus* was found on the chicken farm. *Mam. vitulinus* and *Mam. stepanovicii* were commonly discovered in residential areas.

### 3.5. Population of Macrococcus spp.

**Figure 6:**
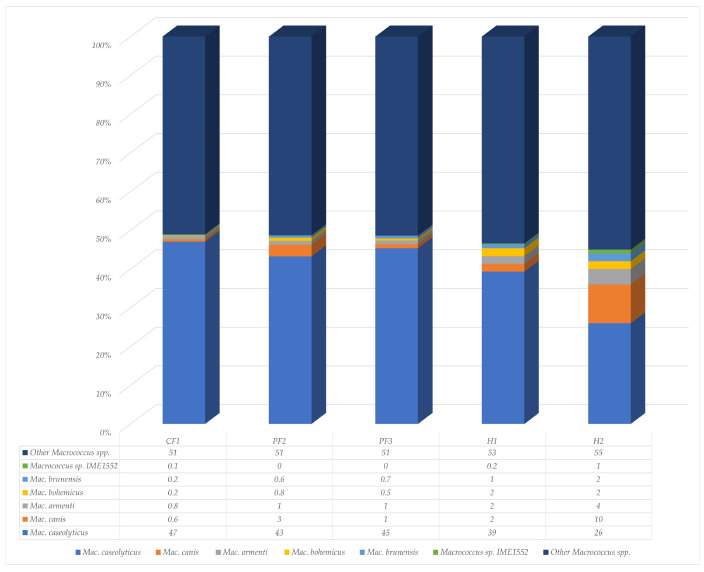
Percentages of species in the genus *Macrococcus* isolated from *M. domestica* collected across one chicken farm (CF1), two pig farms (PF2 and PF3), and two residential areas (H1 and H2) around Walailak University.

Six species of *Macrococcus* were included in the experiment, including *Macrococcus* (*Mac.*) *caseolyticus*, *Mac. canis*, *Mac. armenti*, *Mac. bohemicus*, *Mac. brunensis*, and *Macrococcus* sp. IME1552. The percentage of *Macrococcus* spp. in CF1, PF2, PF3, H1, and H2 was 15, 2, 15, 18, and 0.5, respectively. *Mac. caseolyticus* emerged as the most prevalent species identified in *M. domestica*, accounting for an average of 40% (Figure 6). Most of these species were discovered on chicken farms, and the lowest representation was in residential areas. The percentage of *Mac. canis* was higher than in the other places.

### 3.6. Antimicrobial-Resistant Genes

A list of the genes that were found to be resistant in this investigation is provided in Table 1. In accordance with the classification of antibacterial medications, genes were arranged into the following six categories: aminoglycoside, beta-lactam, trimethoprim, macrolide, lincosamide, streptogramin B (MLS), tetracycline, and fosfomycin. Six aminoglycoside-resistant genes were discovered in CF1. Three beta-lactam-resistant genes were found only in H2. Trimethoprim-resistant genes were found only in CF1, PF2, and H1. The highest variable resistant genes were found on MLS antibiotics, up to 7 genes. Fosfomycin-resistant genes were found only in *M. domestica* within residential areas (H1 and H2).

## 4. Discussion

*Musca domestica* plays a role in the dissemination of antimicrobial bacteria, especially the family Staphylococcaceae, which is a major contributor to severe infections worldwide. This investigation revealed the epidemiology and antimicrobial-resistant genes of three major genera within the family Staphylococcaceae, sourced from *M. domestica* across five Southern Thai places via metagenomic analysis. The variable of genus in this family was observed across the five places, with *Staphylococcus* being the most prevalent genus, while *S. gallinarum* exhibited the highest population within this genus. The highest resistant gene diversity was observed in residential areas because of antimicrobial-resistant genes. This marks the inaugural documentation of the family Staphylococcaceae in Southern Thailand. Although there is no data available for population comparison, our information can be useful for monitoring and managing the distribution of the family Staphylococcaceae between animal farms and households.

The presence of 2837 taxa shared among all five groups indicates the existence of a core microbiota that is crucial for sustaining fundamental ecological functions across various environments or host conditions. Nonetheless, the counts of unique taxa in PF3 (284) and CF1 (525) reflect community variations and suggest that these groups possess unique microbial signatures influenced by particular environmental or host-related factors. The elevated count of distinct taxa in PF3 may stem from microbial diversity or selective pressures particular to that group, while the distinctiveness of CF1 could be associated with house fly-specific or ecological factors [39]. The overlap of 209 taxa between CF1 and H1 indicates a close similarity in the microbial communities of these two groups. This may suggest similar ecological niches, host backgrounds, or environmental exposures [40,41]. The reduced counts of unique taxa in PF2 and H2 (395 and 420 taxa) indicate that the microbial communities in this sample exhibit greater similarity to the other groups. Overall, these results indicate that a conserved set of microbial taxa, known as the core microbiota, persists across all groups. While variation exists at the group level, this contributes to the overall diversity [42,43]. The observed differences could significantly influence ecological dynamics, host–microbe interactions, and the potential functional capabilities inherent to each microbial community [40,44].

The results of this study demonstrated that the fluctuating population of the family Staphylococcaceae was not based on animal farms or residential areas, but rather that the pattern was dependent on individual places. This observation is consistent with recent metagenomic and 16S rRNA studies, which highlight that the house fly microbiome is highly malleable and functions as a biological reflection of the specific local environment and its associated selective pressures [39]. In the cafeteria area, the population of *Staphylococcus* spp. in house flies was higher than in the other places. It is possible that the population of humans in this place was higher than in the others, and then *Staphylococcus* spp., which constitutes part of the normal flora in humans, was commonly found in house flies in this place [45]. Furthermore, the prevalence of specific bacterial groups in these human-dense areas often correlates with a distinct profile of antimicrobial resistance genes (ARGs) linked to human activities, reinforcing the role of flies as local indicators of both microbial diversity and clinical resistance [46].

The most common genus in the Staphylococcaceae family found in *M. domestica* during this investigation was *Staphylococcus*. Consistent with the earlier research, the population of CoNS exceeded that of CoPS [24]. In this study, *S. gallinarum* exhibited the highest population. The high prevalence showed that the potential for cross-contamination might be present during the movement of flies between livestock areas and other environments. The results contrast with a previous study in Turkey, which indicated that *S. gallinarum* was rare [45]. In that experiment, *S. sciuri*, which has now been reclassified and renamed to *Mam. sciuri* within the genus *Mammaliicoccus*, exhibited the highest population among the genus *Staphylococcus* [1,45]. *Mam. sciuri*, also known as *S. sciuri*, stands out as one of the most notable pathogens within the family Staphylococcaceae. Following reclassification, this microorganism has garnered increasing attention due to the majority exhibiting multidrug-resistant properties [47,48,49,50]. Consequently, *Mam. sciuri* has not been documented regarding SCC*mec* or other horizontal gene transfer, yet it harbors numerous resistance genes, complicating antimicrobial treatment in both humans and animals [6,12]. Furthermore, *Mam. sciuri* was frequently identified in the environment, as well as in animals and humans, and it contributes to the presence of opportunistic bacteria in both animals and humans [45,51,52,53].

*S. aureus* is a well-known CoPS that lives on *M. domestica* [22,24], but this is the first time *S. pseudintermedius* has been found in house flies. *S. pseudintermedius* is usually a commensal of animal skin and an opportunistic pathogen in infections in dogs and people [54,55,56]. It has also been found in veterinary settings [57]. Our findings suggest that houseflies could be a new mechanical vector for this bacterium, possibly connecting infected animals to the wider environment. This finding indicates that we must monitor *M. domestica* more closely, as they may facilitate the transmission of *S. pseudintermedius* to previously unidentified host species.

Fosfomycin is a strong antibiotic that destroys microorganisms. It is widely utilized in both human and veterinary treatment, including poultry, livestock, and companion animals [58]. It is still one of the most significant drugs for treating clinical diseases like *P. aeruginosa*, *K. pneumoniae*, and *S. aureus* [58]. Fosfomycin is the first choice for treating severe, multidrug-resistant (MDR) infections in Thailand [59,60]. The identification of resistance genes *fosBx1* and *fosD* compromises their clinical relevance. An increasing number of studies on these genes, which reduce the drug’s efficacy in humans, animals, and food sources [61,62,63]. A concerning pathway of environmental transmission, including these genes, may have been acquired through exposure to anthropogenic waste or proximity to human settlements [64]. We suggested that house flies could serve as bio-indicators for the spread of fosfomycin resistance from urban refuse to the wider environment.

Several limitations of this study warrant consideration. Firstly, the sample collection was conducted over a two-month period rather than a synchronized time point, potentially introducing temporal variability in the microbial profiles due to fluctuating environmental conditions. Secondly, this study analyzed only the microbiota from the external surface of the flies without comparing the internal gut microbiota. Given that these two anatomical sites can harbor distinct bacterial compositions [39], the results represent the total microbial carriage rather than site-specific colonization. To enhance the understanding of the fly-associated microbiome, future research should implement synchronized collection protocols and compare the external surface and the internal gut microbiota.

## 5. Conclusions

The family Staphylococcaceae is one of the important zoonotic bacteria found in *M. domestica*. Transmission through house flies may result in localized illness. This study presents the inaugural report in southern Thailand that mentions the population of the family Staphylococcaceae, encompassing *Macrococcus*, *Mammaliicoccus*, *Staphylococcus*, and others, including potential resistance genes identified within this family. Our findings pertain to the family Staphylococcaceae population in *M. domestica* around residential areas, which exhibited varying multidrug-resistance genes, particularly those resistant to fosfomycin. In this study, our sample was restricted to five locations in southern Thailand. Therefore, further study should increase the study areas, particularly residential areas, to elucidate caution zones.

## Figures and Tables

**Table 1:** Resistant genes in the family Staphylococcaceae were identified in CF1, PF2, PF3, H1, and H2.

Antimicrobial Group	Resistant Genes				
	CF1	PF2	PF3	H1	H2
**Aminoglycoside**	*aadA2*, *ant6-Ia*, *aph(3″)-III*, *apH-Stph, sat4A*, *spw*	*aadA2*, *aph(3″)-III*	*aadA2, ant6-Ia*, *aph(3″)-III*, *sat4A, spw*	*aadA3, aph(3″)-III, sat4A*, *spw*, *aac6-Aph2*	*apH-Stph*, *ant6-Ia*, *aph(3″)-III*, *sat4A*, *spw*
**Beta-lactam**	*blaARL-3*	—	—	—	*blaI*, *mecA1*, *mecI*
**Trimethoprim**	*dfrG*	*dfrE*, *dfrG*	*—*	*dfrE*	—
**Macrolide, Lincosamide and Streptogramin B (MLS)**	*erm(B)*, *erm(C)*, *lin(A)*, *lnu(B)*, *mef(A)*, *mph(C)*, *msr (D)*	*erm(A)*, *erm(B)*, *erm(C)*, *lin(A)*, *lnu(B)*, *cfr(B)*, *Isa(A)*	*erm(B)*, *erm(C)*, *erm(F)*, *Inu(B)*	*erm(B)*, *erm(F)*, *lin(A)*, *Inu(B)*	*erm(B)*, *erm(C)*, *erm(Y)*, *lin(A)*, *lnu(B)*, *msr(A)*, *sal(A)*
**Tetracycline**	*tetK*, *tetL*, *tetM*, *tetR*, *tetW*	*tetL*, *tetM*, *tetR*, *tetS*, *tetW*	*tet(36)*, *tetL*, *tetR*, *tetS*	*tetL*, *tetR*, *tetS*	*tetK*, *tetL*, *tetS*
**Fosfomycin**				*fosBx1*	*FosD*

## Data Availability

The data presented in this study are available on request from the corresponding author.

## Supplementary Materials

The following supporting information can be downloaded at: https://www.mdpi.com/article/10.3390/insects17050461/s1. Table S1: Distribution of Taxa intersections across five sample groups used for UpSet plot analysis. Figure S1: Taxonomic composition and relative abundance within the family Staphylococcaceae visualized using the Krona program: CF1 The hierarchical chart displays the distribution of genera and species, with sector sizes representing their respective percentages. Figure S2: Taxonomic composition and relative abundance within the family Staphylococcaceae visualized using the Krona program: PF2 The hierarchical chart displays the distribution of genera and species, with sector sizes representing their respective percentages. Figure S3: Taxonomic composition and relative abundance within the family Staphylococcaceae visualized using the Krona program: PF3 The hierarchical chart displays the distribution of genera and species, with sector sizes representing their respective percentages. Figure S4: Taxonomic composition and relative abundance within the family Staphylococcaceae visualized using the Krona program: H1 The hierarchical chart displays the distribution of genera and species, with sector sizes representing their respective percentages. Figure S5: Taxonomic composition and relative abundance within the family Staphylococcaceae visualized using the Krona program: H2 The hierarchical chart displays the distribution of genera and species, with sector sizes representing their respective percentages.

## Abbreviations

OTU	Operational Taxonomic Units
HGT	Horizontal gene transfer
AMR	Antimicrobial resistance
ARGs	Antimicrobial resistance genes (ARGs)
CF	Chicken farm
PF	Pig farm
H	Residual areas
TSB	Tryptic soy broth
*S.*	*Staphylococcus*
*Mam.*	*Mammaliicoccus*
*Mac.*	*Macrococcus*
