# Supplementary material for: Epidemiology and Antimicrobial-Resistant Genes of Family Staphylococcaceae in Musca domestica: Case Studies from Chicken Farm, Pig Farms, and Residential Areas in Southern Thailand
Source: Insects. 2026 Apr 28;17(5):461. doi: 10.3390/insects17050461 (PMC13207840; doi:10.3390/insects17050461)
Supplement: Supplementary file 1 [file insects-17-00461-s001.zip › insects-4186056-supplementary.pdf]

Supplementary materials

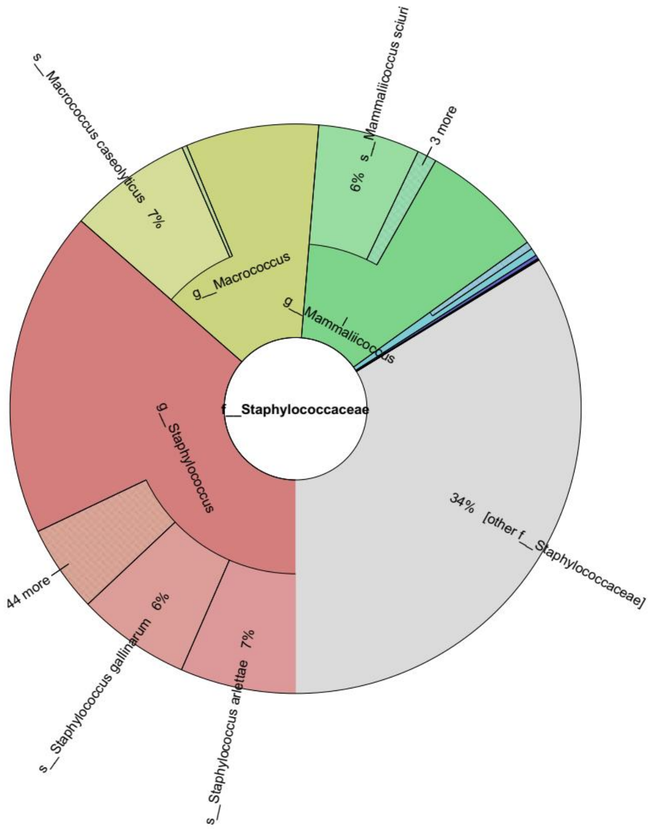

Figure S1. Taxonomic composition and relative abundance within the family Staphylococcaceae visualized using the Krona program: CF1 The hierarchical chart displays the distribution of genera and species, with sector sizes representing their respective percentages.

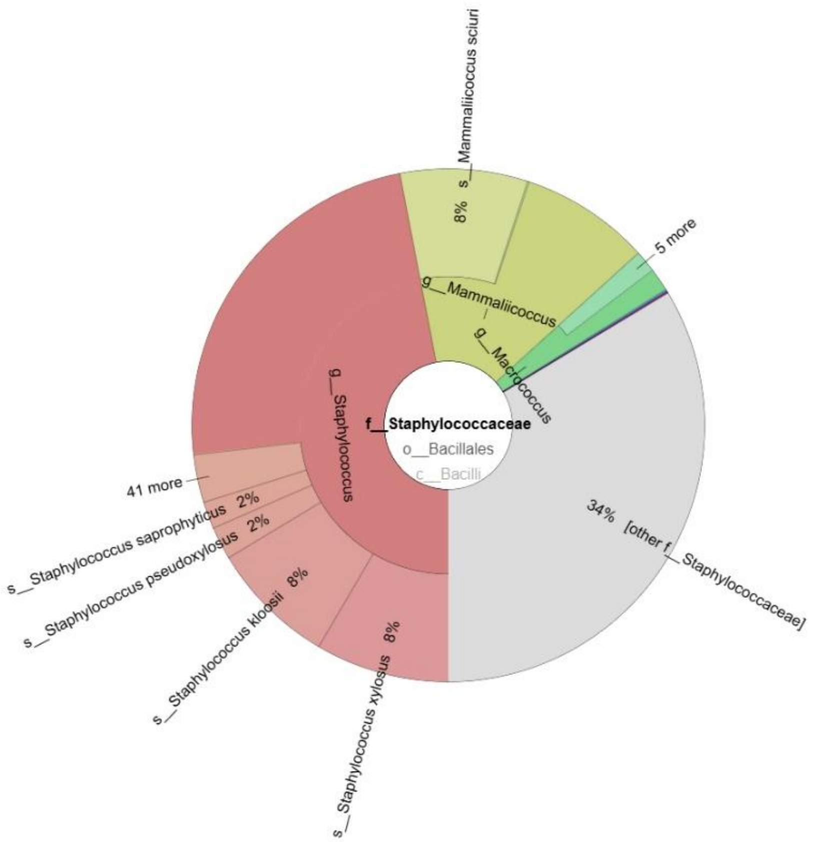

Figure S2. Taxonomic composition and relative abundance within the family Staphylococcaceae visualized using the Krona program: PF2 The hierarchical chart displays the distribution of genera and species, with sector sizes representing their respective percentages.

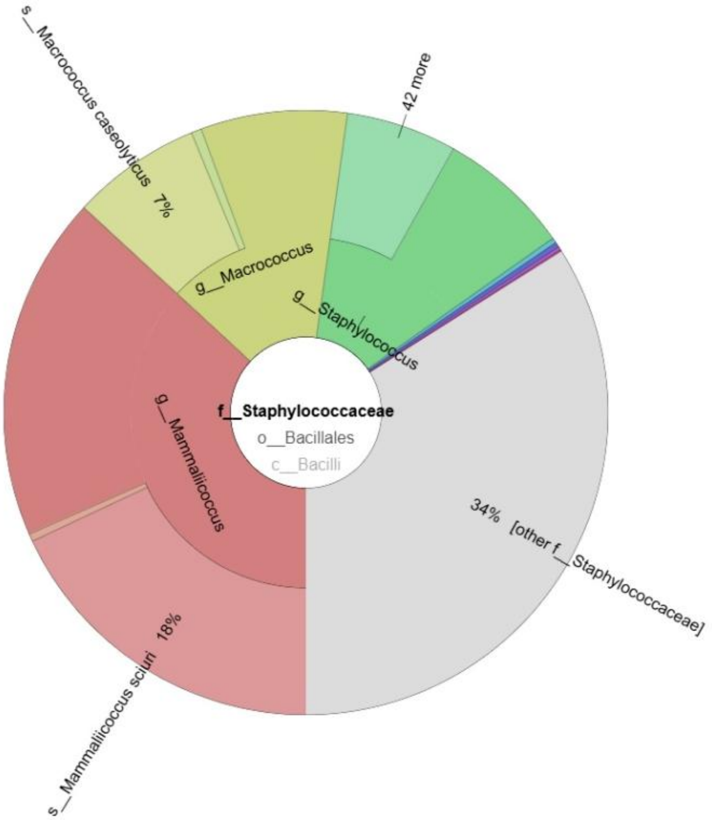

Figure S3. Taxonomic composition and relative abundance within the family Staphylococcaceae visualized using the Krona program: PF3 The hierarchical chart displays the distribution of genera and species, with sector sizes representing their respective percentages.

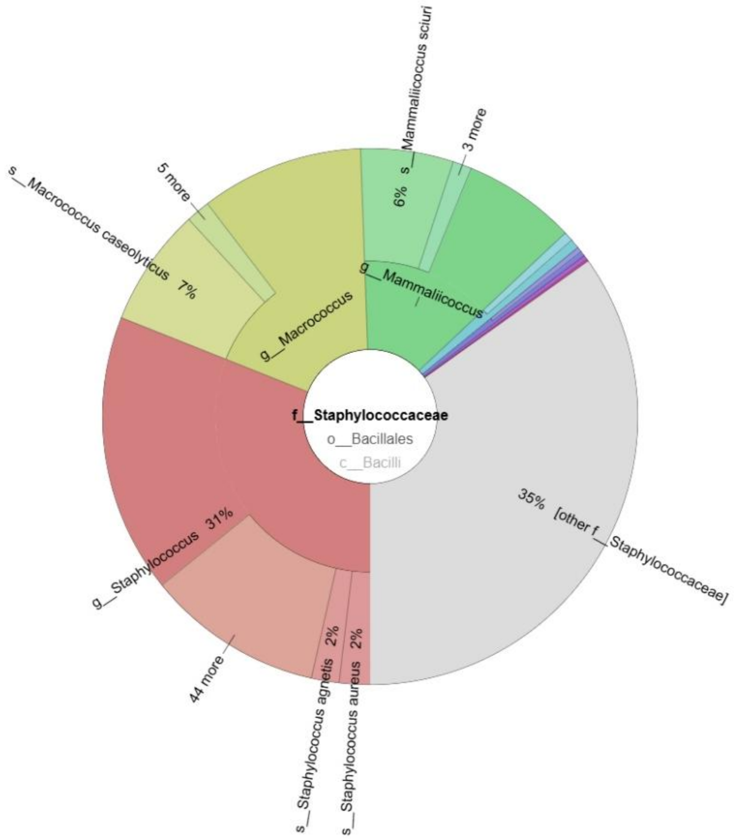

Figure S4. Taxonomic composition and relative abundance within the family Staphylococcaceae visualized using the Krona program: H1 The hierarchical chart displays the distribution of genera and species, with sector sizes representing their respective percentages.

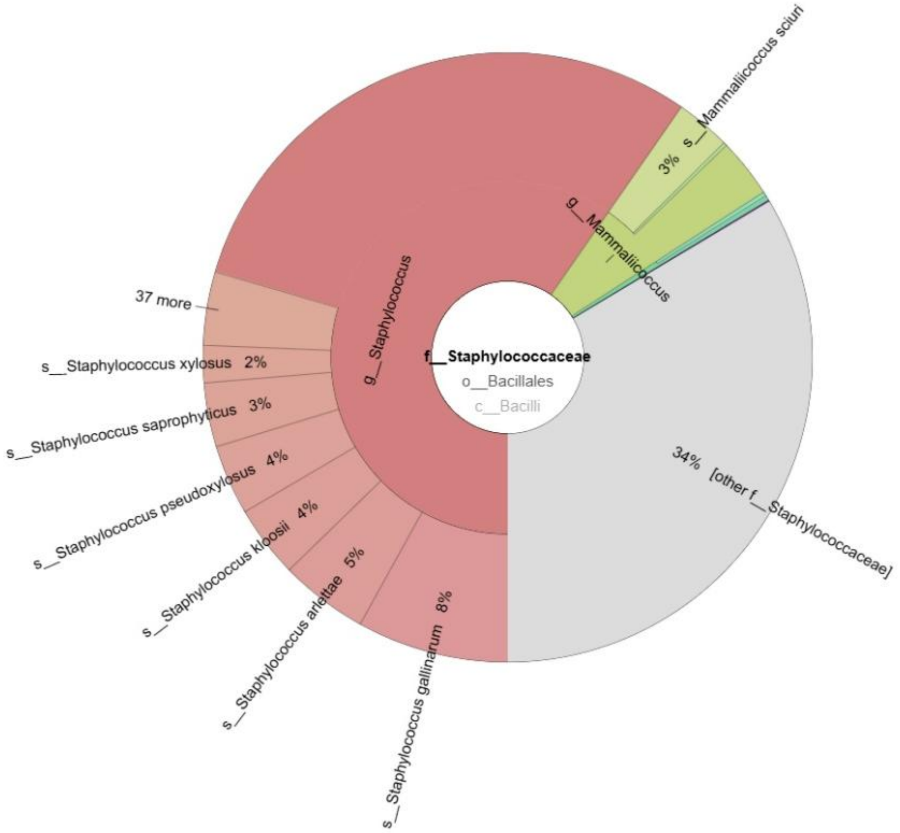

Figure S5. Taxonomic composition and relative abundance within the family Staphylococcaceae visualized using the Krona program: H2 The hierarchical chart displays the distribution of genera and species, with sector sizes representing their respective percentages.

Table S1 Distribution of Taxa intersections across five sample groups used for UpSet plot analysis.

Groups	Num_groups	Count
CF1	1	525
H2	1	420
PF2	1	395
H1	1	356
PF3	1	284
CF1, PF2	2	491
CF1, H1	2	209
CF1, PF3	2	181
H1, PF3	2	168
H2, PF2	2	103
H1, PF2	2	97
CF1, H2	2	96
PF2, PF3	2	91
H2, PF3	2	47
H1, H2	2	43
CF1, PF2, PF3	3	559
CF1, H1, PF3	3	354
CF1, H1, PF2	3	348
CF1, H2, PF2	3	287
H1, PF2, PF3	3	115
H1, H2, PF3	3	75
CF1, H1, H2	3	67
CF1, H2, PF3	3	53
H2, PF2, PF3	3	37
H1, H2, PF2	3	31
CF1, H1, PF2, PF3	4	1414
CF1, H2, PF2, PF3	4	619
CF1, H1, H2, PF2	4	324
CF1, H1, H2, PF3	4	247
H1, H2, PF2, PF3	4	73
CF1, H1, H2, PF2, PF3	5	2837
